# Draft genome sequence of *Leishmania tropica* isolated from a cutaneous leishmaniasis patient in Pakistan

**DOI:** 10.1128/mra.00150-25

**Published:** 2025-12-16

**Authors:** Irshad Ullah, Arsh Bibi, Shumaila Naz

**Affiliations:** 1 Department of Biological Sciences, National University of Medical Sciences (NUMS) 445232, Rawalpindi, Pakistan; 2 Agricultural Biotechnology Division, National Institute for Biotechnology and Genetic Engineering (NIBGE), Constituent College Pakistan Institute of Engineering and Applied Sciences (PIEAS) 194772, Faisalabad, Pakistan; University of California Riverside, Riverside, California, USA

**Keywords:** *Leishmania tropica*, genome sequence, cutaneous leishmaniasis, Pakistan

## Abstract

Cutaneous leishmaniasis (CL) is a major health issue in Pakistan, mainly caused by *Leishmania tropica* (*L. tropica*). Here, we report the draft genome sequence of an *L. tropica* isolate from a CL patient in Pakistan.

## ANNOUNCEMENT

Leishmaniasis is a vector-borne parasitic disease caused by protozoan parasites of the genus *Leishmania* and transmitted through the bite of infected female sand flies (1). Cutaneous leishmaniasis (CL) is the most common form and is endemic in Pakistan, where *Leishmania tropica* (*L. tropica*) and *L. major* are the predominant species (2). Despite its significant disease burden, genomic data on local *Leishmania* isolates from Pakistan remain limited. The availability of whole genome sequences from clinical isolates is essential to enhance our understanding of parasite diversity, drug resistance mechanisms, and transmission dynamics in endemic regions. Here, we report the draft genome sequence for the *L. tropica* isolate obtained from a CL patient in Khyber Pakhtunkhwa (KPK), Pakistan.

A 21-year-old male presenting with an ulcerated lesion on his limbs was clinically suspected of CL and confirmed positive for *Leishmania* by microscopy of Giemsa-stained lesional aspirates. Parasites were cultured in Novy-MacNeal-Nicolle medium at 24–26°C and subsequently expanded in RPMI-1640 medium supplemented with 10% fetal calf serum (3). Genomic DNA was extracted using a commercial kit (Thermo Fisher Scientific, USA), quantified using a Nanodrop and Qubit fluorometer, and checked for integrity by agarose gel electrophoresis. A sequence library was prepared with the Nextera XT kit DNA library preparation kit (Illumina, USA) and sequenced on the Illumina HiSeq platform, generating paired-end reads of 151 bp in mean length.

**Fig 1:**
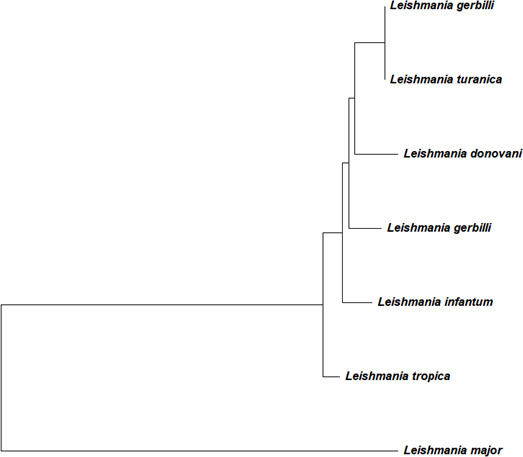
The phylogeny was inferred using the maximum likelihood method and Tamura-Nei model (10) of nucleotide substitutions, and the tree with the highest log likelihood (−10,291.87) is shown.

Whole genome sequencing produced 11.66 million paired-end reads of 151 bp length. FastQC (v0.11.9) confirmed that all reads passed quality control. The average GC content of the raw reads was 61% and provided ~31× genome coverage (4, 5). The raw reads were then trimmed using fastp v0.20.0 and the mean length was reduced to 150 bp from 151 bp. *De novo* genome assembly was performed using SPAdes v3.15.5 with default parameters, producing 26,199 contigs (≥200 bp) and a total genome size of 35.8 Mb. The final statistics of assembly were evaluated using QUAST v5.3.0 (6), which reported a GC content of 59.79%. The N50 value was 4,364, and the largest contig was 55.6 kb. The assembly is fragmented but consistent with the expected genome size and GC content of *L. tropica*. Prior to submission, quality filtering was performed to improve assembly reliability. Short contigs (<200 bp) were removed using Seqkit v2.8.1, a fast and versatile toolkit for processing FASTA/Q files, to eliminate very small sequences that might represent assembly artifacts (7). The resulting assembly was further screened by NCBI using the Foreign Contamination Screen to detect and remove any potential contaminant sequences, resulting in a clean draft genome suitable for downstream analysis (8). Phylogenetic tree analysis was conducted using MEGA12 (9) on the HSP70 coding sequence using the maximum likelihood method, which placed the isolate within the *L. tropica* cluster, confirming species identity (Fig. 1).

The draft genome adds to the limited genomic resources available for *L. tropica* in South Asia and may facilitate comparative analyses and future studies on parasite biology, drug resistance, and epidemiology (11).

## Data Availability

The sequences for the assembly and raw reads have been submitted to NCBI GenBank and SRA under accession number JBMEXS000000000 and PRJNA1314960, respectively. The raw sequencing reads generated in this study have been deposited in the NCBI Sequence Read Archive (SRA) under accession number SRR32992066.
